# Rs10887800 *renalase* gene polymorphism influences the level of circulating renalase in patients undergoing hemodialysis but not in healthy controls

**DOI:** 10.1186/s12882-017-0543-4

**Published:** 2017-04-03

**Authors:** Anna Stec

**Affiliations:** Department of Nephrology, Medical University of Lublin, 8 Jaczewskiego Street, 20954 Lublin, Poland

**Keywords:** Hemodialysis, Polymorphism, Renalase, Renalase gene

## Abstract

**Background:**

Human renalase (RNLS), a recently identified flavoprotein with oxidoreductase activity, is secreted into blood by kidneys and metabolizes circulating catecholamines. Recent studies have revealed that common polymorphisms in *RNLS* gene might affect the risk of several cardiovascular conditions in hemodialyzed patients. However, the exact mechanism underlying this link remains unclear. The study aims to investigate the association between *RNLS* gene polymorphisms and plasma renalase level in ESKD patients undergoing hemodialysis (HD group) and healthy controls (HC).

**Methods:**

A total of 309 hemodialyzed patients and 90 controls were enrolled in the study. All the participants were genotyped for two *RNLS* SNPs (rs2576178 and rs10887800) using PCR-RFLP method. Plasma renalase concentrations were determined by enzyme-linked immunosorbent assay (USCN Life Science Inc., Wuhan, China). The IBM SPSS Statistics for Windows, version 20 (IBM Corp., Armonk, NY, USA) was used for statistical analyses.

**Results:**

Genotype distribution and allele frequencies of studied SNPs did not differ between two analyzed groups, *p* > .050. RNLS concentration in HD group (33.54 μg/mL) was significantly higher than in HC (13.16 μg/mL), *p* < .001. HD patients with rs10887800AA genotype had lower renalase level (29.32 μg/mL) compared to those with AG (34.52 μg/mL), *p* < .010 and GG genotype (35.91 μg/mL), *p* < .010. No significant differences in plasma RNLS between rs10887800AG and GG carriers were observed, *p* > .050. Interestingly, in HC group rs10887800 polymorphism did not influence RNLS concentration. Rs2576178 SNP did not affect the level of plasma RNLS either in HD group or in HC.

**Conclusion:**

Rs10887800 polymorphic variant of *RNLS* gene influences the level of circulating RNLS in patients undergoing hemodialysis, and thus elucidates the potentially functional relevance of this polymorphism in HD population.

## Background

Human renalase (RNLS), a recently identified flavoprotein with oxidoreductase activity, is secreted into blood mainly by kidneys and metabolizes circulating catecholamines [1, 2]. By the regulation of the sympathetic tone, RNLS modulates cardiac functions and systemic blood pressure [3]. Experimental data indicate that, apart from its enzymatic activity, RNLS may also act as a cytokine and manifest cytoprotective and immunomodulary properties [4, 5]. Seven isoforms of RNLS (hRenalase 1–7) have been identified so far. hRenalase 1, a 342 amino acids protein with a molecular weight of ~ 37.8 kDa, is the most highly expressed form [6]. The renalase gene (*RNLS*) is positioned on chromosome 10 (10q23.31), has 13 exons and several polymorphic variants [7]. The recent studies on hemodialyzed population have revealed that single nucleotide polymorphisms (SNPs) of *RNLS* gene affect the risk of cardiovascular conditions such as arterial hypertension (HY) or coronary artery disease (CAD) [8, 9]. However, the exact mechanism underlying the functional interplay between those pathologies and *RNLS* genetic variants remains unknown. To clarify the functional relevance of *RNLS* polymorphisms we performed a study that aimed to investigate whether two SNPs of the *RNLS* gene (rs10887800 and rs2576178) influence plasma RNLS concentration in hemodialyzed patients and healthy controls.

## Methods

A total of 399 participants, including 309 end-stage kidney disease (ESKD) patients (HD group) and 90 healthy controls (HC group) were consequtively enrolled in the study from January 1, 2012 until December 31, 2013. There were more females (53.64%) than males (46.36%). The mean age of the study population was 59.25 ± 11.32 years. All subjects were Caucasians of Polish origin. The most common cause of ESKD was diabetic kidney disease (23%). The remaining reasons were as follows: chronic glomerulonephritis (18.2%), hypertensive kidney disease (12.6%), autosomal dominant polycystic kidney disease (10%), chronic tubulo-interstitial nephritis (9.1%), obstructive nephropathy (8.4%) and others or undefined (18.7%). Patients with ESKD were recruited from five hemodialysis (HD) units in eastern Poland. All patients received standard bicarbonate hemodialysis with low-flux synthetic membranes thrice-weekly for 3–5 h per session. At the beginning of the dialysis blood samples for renalase measurements and genetic research were drawn from the venous side of the vascular access into the EDTA (ethylenediaminetetraacetic acid) containers. Socio-demographic and clinical data were collected by nephrologists through interview, patient’s medical records analysis and physical examination. Adequacy of hemodialysis treatment in all HD patients was assessed by the calculator, which uses the Daugirdas II equation formula for Single Pool Kt/V for urea (spKt/Vurea).

Healthy controls group was obtained from volunteers (hospital workers or blood donors) without history of chronic diseases. According to the Declaration of Helsinki all participants signed the informed consent document. The study protocol was approved by the local Ethics Committee.

### The plasma renalase measurement

The blood samples were taken from all individuals into the EDTA tubes. Plasma was separated and stored at −80 °C until the assay. Renalase concentrations were determined by commercially available enzyme-like immunosorbent assay (ELISA) kits from USCN Life Science Inc. (Wuhan, China).

### DNA isolation and genotyping

In the study two renalase gene polymorphisms (rs2576178 in 5′ flanking region and rs10887800 in intron 6) were genotyped using PCR-RFLP (polymerase chain reaction-restriction fragment length polymorphism) technique. Genomic DNA was obtained from peripheral blood leukocytes in accordance with the modified protocol of Madisen et al. [10]. Primers sequences used for analysis were as follows: rs10887800 SNP sence 5′-CAGGAAAGAAAAGAGTTGACAT-3′ antisense 5′-AAGTTGTTCCAGCTACTGT-3′ and rs2576178 SNP sense 5′-AGCAGAGAAGCAGCTTAACCT-3′ antisense 5′-TTATCTGCAAGTCAGCGTAAC-3′. DNA amplification was carried out in a PTC 200 Thermal Cycler (MJ Research, Inc. Waltham, MA). All reagents were from MBI Fermentas (St. Leon-Rot, Germany). The 30 μl of final volume contained: 300 ng DNA, 1 μM of each primer, 200 μM each dNTP, 1.5 mM MgCl_2,_ 50 mM KCl, 10 mM Tris-HCl buffer (pH 8.3) and 2 U Taq polymerase. The reaction conditions were: initial denaturation at 95 °C for 6 min followed by 35 cycles of denaturation at 94 °C for 30s, annealing at 60 °C for 30s, extension at 72 °C for 1 min and the final extension for 7 min. The 10 μl of PCR products were digested at 37 °C for 6–10 h with 5 U of Pst I and Msp I restriction endonucleases, then separated by 2.5% agarose gel electrophoresis and finally identified with ethidium bromide staining.

### Statistical analysis

The IBM SPSS Statistics software package (version 20, IBM Corp., Armonk, NY, USA) was applied for data analysis. A two-tailed *p*-value < .050 was regarded as statistically significant. Hardy-Weinberg equilibrium was tested using the conventional *χ*2 goodness-of-fit test. The distribution of genotype and the allele frequency were calculated by a 2 × 2 contingency *χ*2 test. The means of continuous variables were compared between two independent study groups by a student’s *t* test, whereas discrete variables by a *χ*2 test of independence.

Plasma renalase concentrations were compared between three genotype-based subgroups by analysis of variance (ANOVA). The Bonferroni test was used for post-hoc analyses. Subsequently, analysis of covariance (ANCOVA) was conducted to assess the influence of genotypes on RNLS level after adjustment for potentially confounding effect of age, sex, BMI, albumin and hemoglobin level, the presence of hypercholesterolemia and residual diuresis. Sidak’s correction test was used for post-hoc analyses.

## Results

**Fig. 1:**
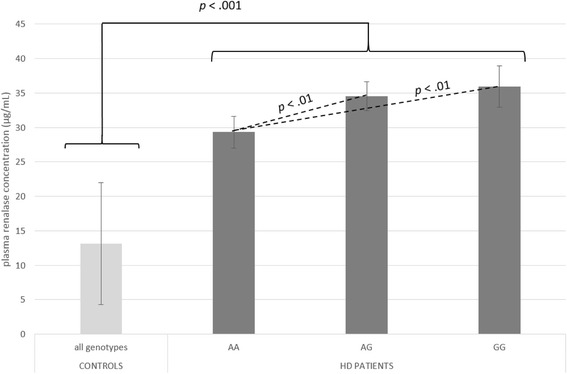
Plasma renalase concentrations as a function of rs10887800 renalase gene polymorphism

All the participants were genotyped for two *RNLS* gene polymorphisms: rs10887800 (intron 6) and rs2576178 (5′flanking region) and their plasma RNLS concentrations were measured. Demographic and clinical characteristics of the study groups are summarized in Table 1. Compared with controls HD patients were older (64.1 ± 14.1 years vs 42.4 ± 11.3 years, *p* < .001) and more frequently male (50.8% vs 31.1%, *p* < .001). They also have higher mean plasma RNLS level (33.54 ± 12.37 μg/mL vs 13.16 ± 4.5 μg/mL *p* < .001). The genotypes distribution in all studied groups was in Hardy-Weinberg equilibrium, *p* > .050. The prevalence of genotypes and allele frequencies for both SNPs did not differ between HD patients and HC, *p* < .050. As shown in Table 2 among HC group no genotype-based differences in mean plasma RNLS levels were observed, whereas such variations were noticed in HD population (Table 3). HD patients with rs10887800AA genotype had significantly lower RNLS level (29.32 ± 9.89 μg/mL) compared to those with AG (34.52 ± 13.12 μg/mL), *p* < .010 and GG genotype (35.91 ± 12.07 μg/mL), *p* < .010. Homozygotes rs10887800GG and AG carriers did not differ in terms of mean plasma RNLS concentrations, *p* > .050 (Fig. 1). In contrast, rs2576178 SNP did not affect the level of plasma renalase in this population. Moreover, in HD group the differences in RNLS levels between genotypes didn’t change statistically after adjustment for potentially confounding effect of age, sex, BMI, albumin and hemoglobin level, the presence of hypercholesterolemia and residual diuresis. Thus, mean plasma RNLS levels in HD patients with rs2576178AA, AG and GG genotypes were: 33.33 ± .913, 33.59 ± 1.122, 35.26 ± 2.541 μg/mL, respectively, *p* > .050. Whereas, mean plasma RNLS levels in HD patients with rs10887800AA, AG and GG genotypes were: 28.77 ± 1.324, 34.32 ± .910, 37.12 ± 1.424 μg/mL, respectively, *p* < .010.

**Table 1:** Demographic and clinical characteristics of studied groups

Variables	HD patients (*n* = 309)	Controls (*n* = 90)	*p* value*
Age (years)	64.1 ± 14.1	42.4 ± 11.3	<.001
Sex (Male/Female)	157/152	28/62	<.001
BMI (kg/m^2^)	25.8 ± 5.3	25.3 ± 4.0	>.050
spKt/Vurea	1.39 ± .62	NA	
Plasma renalase (μg/mL)	33.54 ± 12.37	13.16 ± 4.5	<.001
Rs10887800			
GG/AG/AA	71/161/77	16/49/25	>.050
G *vs* A	.49/.51	.45/.55	>.050
Rs2576178			
GG/AG/AA	23/118/168	7/33/50	>.050
G *vs* A	.27/.73	.26/.74	>.050

Continuous variables are presented as means ± SD (standard deviation). Discrete variables are presented as numbers. HD; hemodialyzed, BMI; Body Mass Index, spKt/V; single-pool Kt/V for urea, NA; not applicable, *t-student or *χ*2 tests

**Table 2:** Clinical characteristics of healthy controls classified according to the *renalase* gene polymorphisms

Variables	POL	Genotypes			*p* value*
		AA	AG	GG	
Age (years)	1	42.52 ± 10.52	42.06 ± 13.21	43.13 ± 8.51	>.050
	2	42.52 ± 11.54	42.18 ± 11.28	42.87 ± 11.82	>.050
Sex (Male/Female)	1	15/35	11/22	2/5	>.050
	2	10/15	13/36	5/11	>.050
BMI (kg/m^2^)	1	25.59 ± 3.76	24.42 ± 3.94	27.27 ± 5.54	>.050
	2	25.65 ± 3.24	25.30 ± 4.46	24.69 ± 3.76	>.050
Plasma renalase (μg/mL)	1	12.80 ± 4.54	13.46 ± 4.81	14.33 ± 2.54	>.050
	2	11.88 ± 4.46	13.63 ± 4.60	13.73 ± 4.16	>.050

Continuous variables are presented as means ± SD (standard deviation). Discrete variables are presented as numbers. POL; polymorphism, 1-rs2576178, 2-rs10887800, BMI; Body Mass Index. *ANOVA or *χ*2 tests

**Table 3:** Clinical characteristics of hemodialyzed patients classified according to the *renalase* gene polymorphisms

Variables	POL	Genotypes			*p* value*
		AA	AG	GG	
Age (years)	1	64.33 ± 13.26	64.25 ± 14.97	61.69 ± 15.20	>.050
	2	65.51 ± 15.07	63.94 ± 13.37	62.95 ± 14.51	>.050
Sex (Male/Female)	1	89/79	62/56	6/17	<.050
	2	40/37	78/83	39/32	>.050
BMI (kg/m^2^)	1	25.50 ± 4.99	25.80 ± 4.64	27.53 ± 9.37	>.050
	2	25.62 ± 4.62	25.45 ± 5.13	26.65 ± 6.31	>.050
Albumin (g/dL)	1	3.94 ± .39	3.93 ± .35	3.88 ± .48	>.050
	2	3.95 ± .35	3.97 ± .38	3.84 ± .41	>.050
Hemoglobin (g/dL)	1	10.89 ± 1.49	10.90 ± 1.30	10.36 ± 1.22	>.050
	2	10.91 ± 1.33	10.96 ± 1.43	10.56 ± 1.42	>.050
Dialysis time (years)	1	6.20 ± 6.46	6.27 ± 5.68	7.65 ± 8.13	>.050
	2	5.50 ± 5.49	6.63 ± 6.24	6.59 ± 7.23	>.050
CHOL, n (%)	1	88 (52.38)	57 (48.30)	10 (43.47)	>.050
	2	39 (50.64)	83 (51.55)	33 (46.47)	>.050
spKt/Vurea	1	1.38 ± .62	1.39 ± .51	1.36 ± .43	>.050
	2	1.37 ± .60	1.37 ± .63	1.39 ± 35	>.050
RD, n (%)	1	67 (39.88)	48 (40.67)	14 (60.86)	>.050
	2	28 (36.36)	61 (37.88)	40 (56.33)	<.050
Renalase (μg/mL)	1	33.31 ± 13.01	33.98 ± 11.22	33.09 ± 13.48	>.050
	2	29.32a ± 9.89	34.52b ± 13.12	35.91b ± 12.07	<.010

Continuous variables are presented as means ± SD (standard deviation). Discrete variables are presented as numbers and percentages (in parentheses). POL; polymorphism, 1-rs2576178, 2-rs10887800, BMI; Body Mass Index, CHOL; hypercholesterolemia, spKt/Vurea; single-pool Kt/V for urea, RD; residual diuresis. *ANOVA or *χ*2 tests.

## Discussion

In the present study, we observed for the first time that rs10887800 polymorphism of the *RNLS* gene (located within intron 6) influences plasma RNLS level in hemodialyzed population. Interestingly, such effect was not observed in healthy controls. HD patients with rs10887800AA genotype had significantly lower RNLS concentration compared to AG and GG carriers. Furthermore, rs10887800AG heterozygotes manifested intermediate values of plasma RNLS, therefore we have suspected a gene dose effect. Obtained results support the hypothesis that rs10887800 polymorphism plays a functional role in *RNLS* gene regulation process. However, it remains unclear whether this intronic SNP exerts a direct impact on *RNLS* gene expression or whether instead it is in linkage disequilibrium with another functional SNP. Likewise, it is still unknown why the effect of rs10887800 polymorphism on RNLS level varied across different population groups. It could be modulated by enviromental components associated with ESKD or hemodialysis procedure (e.g., increased oxidative stress, sympathetic hypractivity) but the role of other regulatory mechanisms should be considered. Recent studies have revealed that several transcription factors (viz. Sp1, STAT3 and ZBP 89) play a crucial role in the regulation of *RNLS* gene transcription [11]. In addition, it was demonstrated that nicotine, dopamine and epinephrine activate *RNLS* gene expression in various cell types [12, 13]. Furthermore, studies by Kalyani et al. provided an evidence for post-transcriptional regulation of both mouse and human *RNLS* genes by miR-29 and miR-146 microRNAs [14].

As far as the authors know no other papers concerning the influence of *RNLS* gene variants on plasma RNLS concentrations have been published. Data from genome-wide association studies (GWAS) have revealed the link between rs10749571 polymorphism (3′ untranslated region of *RNLS* gene) and both glucose and triglyceride levels in a large human population, thereby suggesting the association of this SNP with cardiometabolic traits [15, 16]. Rezk et al. in a study of 178 Egyptian patients with chronic kidney disease (CKD) and 178 healthy controls have demonstrated a higher level of norepinephrine among CKD patients with CC genotype of rs2296545 SNP (exon 2 of *RNLS* gene) compared to GG and GC carriers. Similarly to our results, polymorphic effect was not observed in healthy controls [17].

To date, only a few papers have shown *RNLS* SNP genotyping results in hemodialyzed populations. Kiseljakovic et al. in a study of HD patients from Bosnia and Herzegovina have reported higher G allele frequency of rs2576178 polymorphism compared to our results (0.44 vs 0.27), but similar to the one observed by Ahlawat et al. in a CKD population from North India (0.5) [18, 19]. The descrepancy between presented results could be attributed to ethnic differences. Meanwhile, the prevalence of G allele of rs10887800 SNP in our study was comparable to the one in Chinese hypertensive population described by Zhao et al. (0.49 vs 0.5) [7].

Our present study has revealed significantly higher level of plasma RNLS in HD population compared to healthy controls. Consistent results were obtained by other researchers for patients undergoing both hemodialysis [20–23] and peritoneal dialysis [24]. In all those studies RNLS level was determined by commercially available ELISA kit (USCN Life Science Inc., Wuhan, China) with monoclonal antibodies specific to hRenalase1 isoform. Interestingly, the opposite results were obtained by investigators using western blot method with polyclonal antibodies [1, 13]. To clarify discrepancy between results obtained by two different methods it was assumed that ELISA-based assay preferentially detects multimers of RNLS which are likely to be formed in ESKD. Furthermore, increased RNLS concentration in advanced CKD patients could be due to cross-reactivity with breakdown products of plasma RNLS, oversecretion of RNLS by other tissues, i.e., cardiomyocytes, liver or skeletal muscles in a presence of kidney failure, sympathetic hyperactivity and decreased RNLS clearance [25].

Our recent studies of HD population have demonstrated the association between *RNLS* gene polymorphisms and the increased risk of cardiovascular diseases [8, 9], but the exact mechanism underlying this link has remained unknown. On the basis of current findings we presume that rs10887800 polymorphism of *RNLS* gene can modify the risk of different human diseases due to modulation of plasma RNLS level. However, since the mechanism of RNLS action is still largely undefined, the extended interpretation of our results should be cautious. Furthermore, previously published data on the pathophysiological role of RNLS have provided contrary results. For instance, in our recent paper rs10887800GG genotype (e.i., related to the higher level of RNLS) was shown to increase the risk of CAD [9], whereas in a study of He et al. plasma RNLS concentration has reversly correleted with the CAD severity [26].

Some limitations should be considered in the interpretation of our findings. Firstly, the study population involved only Caucasian individuals. Therefore, the results may not be generalized to other ethnic groups. Secondly, we did not assess the level of RNLS in urine and dialysis fluid, which could clarify obtained results. Furthermore, the plasma RNLS activity and its intracellular expression were not evaluated. In addition, RNLS measurement method used by us needs further improvements, particularly in the area of better standarization in a population with ESKD. Finally, the in-depth analyses of the impact of rs10887800 polymorphism on RNLS level in subgroups of patients with particular human diseases are needed. However, this is a subject of our ongoing studies.

## Conclusion

The present study suggests for the first time that rs10887800 polymorphism of the *RNLS* gene influences the level of circulating RNLS and thus elucidates its functional relevance in hemodialyzed population. Even though many questions remain open, obtained results provide an important insight into probable mechanisms by which *RNLS* gene variants affect the risk of human diseases. Further investigations on a larger size sample and other ethnic groups are neccesary to confirm and elaborate on our novel findings.

## Abbreviations

ANCOVA: Analysis of covariance
ANOVA: Analysis of variance
CAD: Coronary artery disease
CKD: Chronic kidney disease
EDTA: Ethylenediaminetetraacetic acid
ELSA: Enzyme-like immunosorbent assay
ESKD: End stage kidney disease
HC: Healthy control
HD: Hemodialysis
HY: Hypertension
PCR: Polymerase chain reaction
RFLP: Restrition fragments length polymorphism
RNA: Ribonucleic acid
RNLS: Human renalase
SNP: Single nucleotide polymorphism
